# Monitoring Serum Spike Protein with Disposable Photonic Biosensors Following SARS-CoV-2 Vaccination

**DOI:** 10.3390/s21175857

**Published:** 2021-08-31

**Authors:** John S. Cognetti, Benjamin L. Miller

**Affiliations:** 1 Departments of 1Biomedical Engineering, University of Rochester, Rochester, NY 14627, USA; john.cognetti@rochester.edu; 2 Biochemistry and Biophysics, University of Rochester, Rochester, NY 14627, USA; 3 Optics, University of Rochester, Rochester, NY 14627, USA; 4 Dermatology, University of Rochester, Rochester, NY 14627, USA

**Keywords:** ring resonator, SARS-CoV-2, passive microfluidics, vaccination

## Abstract

While mRNA vaccines have been well-studied in vitro and in animals prior to their use in the human population during the Covid-19 pandemic, their exact mechanisms of inducing immunity are still being elucidated. The large-scale collection of data necessary to fully understand these mechanisms, and their variability across heterogeneous populations, requires rapid diagnostic tests that accurately measure the various biomarkers involved in the immune response following vaccination. Recently, our lab developed a novel “Disposable Photonics” platform for rapid, label-free, scalable diagnostics that utilizes photonic ring resonator sensor chips combined with plastic micropillar cards able to provide passive microfluidic flow. Here, we demonstrate the utility of this system in confirming the presence of SARS-CoV-2 spike protein in the serum of recently vaccinated subjects, as well as tracking a post-vaccination rise in anti-SARS-CoV-2 antibodies. A maximum concentration in SARS-CoV-2 spike protein was detected one day after vaccination and was reduced below detectable levels within 10 days. This highlights the applicability of our rapid photonic sensor platform for acquiring the data necessary to understand vaccine mechanisms on a large scale, as well as individual patient responses to SARS-CoV-2 mRNA vaccines.

## 1. Introduction

The astonishingly rapid development and rollout of the SARS-CoV-2 mRNA-based vaccines in late 2020 and early 2021 has helped tremendously in curbing the Covid-19 pandemic. Current vaccines have demonstrated excellent capabilities in generating SARS-CoV-2-specific antibodies, as well as B- and T- cells that confer long-term immunity [1,2]. However, a detailed population-level understanding of the physiological responses provoked by mRNA-based vaccination against SARS-CoV-2 leading to these desired endpoints remains incomplete. In addition to the scientific importance of such knowledge, it is also potentially an important component of decreasing vaccine hesitancy among the unvaccinated.

Well-established historical vaccines rely on several methods of inducing the production of antibodies in humans, including weakened or killed viruses (respiratory syncytial virus [3], influenza [FluMist^®^] [4]), protein toxins (tetanus [5]), or DNA vectors (Zika [6]). The novel mRNA-based method utilized by the Pfizer/BioNTech and Moderna vaccines, however, makes use of a new method of immune stimulation. These vaccines rely on the uptake of lipid nanoparticles (LNPs), which contain mRNA coding for the full spike protein of SARS-CoV-2. Host cells near the injection site (usually dendritic cells [7]) take up the LNPs, and start translating the mRNA within them into spike protein. These spike proteins then elicit an immune response from T cells [2] and B cells [8], resulting in the antibody production that is well documented in the literature. However, the fate of the spike proteins after they are produced by host cells is unclear, and whether they reach systemic circulation has not been well-studied. The confirmation of viral spike proteins’ presence in the blood, as well as their concentration and time course, is important for understanding the immune response mechanisms following inoculation with this new type of vaccine. Additionally, off-target effects of isolated spike proteins have not been studied in humans, particularly with respect to mRNA vaccines. It has been demonstrated that SARS-CoV-2 spike protein subunit S1 when injected into systemic circulation in rats, disrupts the blood-brain barrier [9]. While this was conducted at a relatively high concentration, it is important to know then what sort of concentrations of spike protein exist in the blood of vaccinated subjects and for how long. A recent study determined that SARS-CoV-2 spike protein exists in the blood after vaccination [10]. While this represents a useful first step, additional verification of these data is required, along with the development of a platform that can determine this on a broader scale.

To address this need, we have developed a photonics-based rapid diagnostic platform that has been demonstrated in its ability to rapidly sense the presence of SARS-CoV-2 receptor binding domain (RBD)-specific antibodies [11]. Photonic integrated circuits (PICs) utilizing resonance-based refractive index sensors are sensitive and can be produced at a wafer scale. Here we incorporated ring resonator sensors, which consist of circular photonic waveguide structures, situated in close proximity (<1 μm) to a bus waveguide, that resonate at specific wavelengths based on their geometry and the refractive index of their environment, with a microfluidic platform that allows for rapid screening of patient fluid samples. When functionalized with specific antibodies (or antigens, when trying to detect specific antibodies circulating in the blood), they have been shown to sensitively detect specific protein markers under microfluidic flow [12]. Recently, our group has integrated these PICs into a disposable platform utilizing passive microfluidics [11]. This platform addresses the need for rapid acquisition of the data needed to understand infectious diseases such as Covid-19 at the necessary scale.

Here, we demonstrate the use of this rapid, inexpensive photonic sensor platform to quantitatively measure the presence of both SARS-CoV-2 full spike protein and anti-SARS-CoV-2 RBD antibodies in the serum of vaccinated subjects in just 3 min. We anticipate that these results will enable broader studies important for understanding the immune response to mRNA-based vaccines. Our platform also provides the potential for rapid detection and monitoring of subject-specific off-target effects in vaccinated individuals.

## 2. Materials and Methods

### 2.1. Photonic Ring Resonator Sensor Chip Design, Fabrication, and Functionalization

Silicon nitride photonic ring resonator sensor chips were designed and fabricated at AIM Photonics as described previously [11]. The chips consisted of a bus waveguide coupled to 2 ring resonators of slightly different diameters, such that they have a predictably spaced spectral resonance. The light was coupled into the bus waveguide using vertical grating couplers, as previously described [11]. The chips were fabricated at the AIM Photonics 300 mm foundry in Albany, NY, using proprietary photolithographic procedures. Briefly, a silicon nitride waveguide layer (about 220 nm thick) was deposited on top of about 5 microns of oxide and photolithographically patterned, with about 5 microns of oxide then deposited above. The top oxide was then etched away down to the level of the nitride, using a mask to selectively expose the ring resonator sensors, such that they may interact with the environment. This leaves a 5 micron-deep trench around each ring, allowing for access by antibody/antigen solutions to be spotted specifically on each ring, described below.

**Figure 1:**
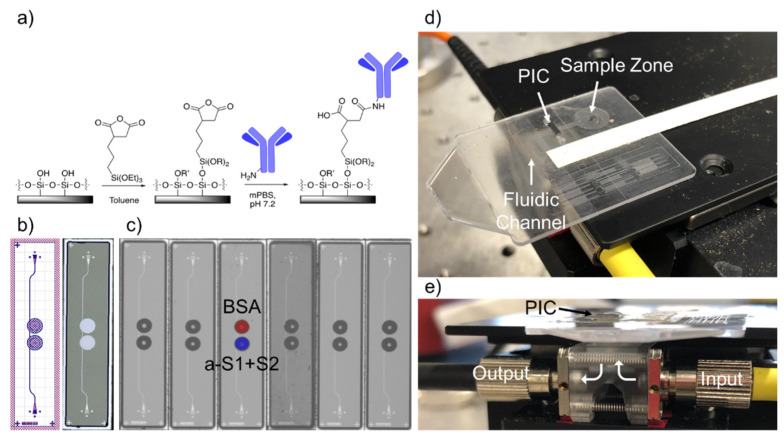
Disposable Photonic Assay Platform. (**a**) Functionalization schematic. The silicon dioxide surface of the chip is functionalized with 3-(triethoxysilyl)propyl succinic anhydride in anhydrous toluene, which then forms an amide bond with free amine groups of antibodies in solution. Here R = H or OEt; R’ = H or 3-succidimidylpropyl trialkoxysilane, as the density is unknown. (**b**) GDS (Graphic Design System) and laser confocal image of the 1 × 4 mm photonic chips used for this study. Two photonic ring resonators are coupled to a single bus waveguide. The trench etched from the oxide cladding around each ring is visible in white on the right. Light is coupled into and out of the PICs via grating couplers, seen centered at the top and bottom of each chip. (**c**) Image of antibody/antigen solutions spotted on chips with a Scienion SX microarrayer. Spots are approximately 300 nL each and are highly reproducible chip-to-chip. The top ring is spotted with 0.1% BSA solution (control) and the bottom ring with 650 μg/mL anti-S1+S2 polyclonal antibody solution. (**d**) Image of the full microfluidic/photonic chip assembly. The sample is pipetted onto the sample zone and wicked through the fluidic channel across the PIC. Whatman paper is situated at the end of the channel for controlled wicking of the full sample volume. (**e**) Side view of the custom optical hub. Infrared light from a tunable laser is coupled vertically into and out of the photonic chip from below. Holes in the disposable micropillar fluidic card allow coupling into the grating couplers of the PIC.

Once received from the foundry, chips were then functionalized to sense SARS-CoV-2 spike protein and anti-SARS-CoV-2 antibodies. First, chips were cleaned with a 50/50 mixture of methanol and hydrochloric acid, rinsed 3x with Nanopure water, and dried with nitrogen. Then they were incubated for 40 min in 1% 3-(triethoxysilyl)propyl succinic anhydride (Gelest, Morrisville, PA, USA) in anhydrous toluene, rinsed for 5 min in pure anhydrous toluene, dried with a stream of nitrogen, and heated at 110 °C for 30 min to allow complete annealing of the functional monolayer and to evaporate any residual toluene. A schematic of the functional chemistry is shown in Figure 1a.

After the sensor chips had been silanized and dried, antibody/antigen solutions were printed on the rings using a Scienion SciFLEXarrayer SX piezoelectric microarrayer equipped with a PDC-60 capillary. The arrayer produced spots of varying sizes depending on the capillary size and the electrical settings on the piezoelectric controller. The number of spots was chosen to yield a total volume of about 3 nL. For this nozzle and these antibody/antigen solutions, 10 spots of approximately 300 pL drops were spotted into each ring. This resulted in the antibody/antigen solution completely filling the trench around each ring without spilling into neighboring rings (Figure 1c). One ring was spotted with 0.1% BSA, as a control for non-specific binding. The test ring was spotted with either anti-SARS-CoV-2 RBD polyclonal antibody (Sino Biological, Wayne, PA; 650 μg/mL) to detect the spike protein or SARS-CoV-2 RBD (Sino Biological, Wayne, PA; 400 μg/mL) to detect antibodies. Both solutions were diluted in modified (i.e., potassium-free) phosphate-buffered saline (mPBS) at pH 7.2. After allowing the antibodies to covalently attach to the rings for 30 min, 20% StabilCoat (Surmodics, Inc., Edin Prairie, MN, USA) solution in assay wash buffer (AWB—mPBS with 3 mM EDTA and 0.01% Tween-20) was overspotted as a stabilizer thus that the chips could be stored dry indefinitely prior to running an assay.

### 2.2. Vaccinated Subject Sample Acquisition

Serum was obtained from 4 vaccinated subjects according to a human subjects research protocol approved by the University of Rochester Medical Center Institutional Review Board. Subjects were aged 25–35 and gave informed consent. All subjects received the Pfizer/BioNTech vaccine (BNT162b2). Timepoints for blood draws were based on individual subject availability, limiting the scope of repeated measurements. Most data were acquired immediately before and for over a month following the second dose, with 2 subjects also providing first-dose timepoints.

Following the draw, whole blood was allowed to clot for 1 hour. Samples were then spun at 1200× *g* for 5 min. The supernatant (serum) was pipetted off and transferred to a fresh centrifuge tube and spun again for 10 min. Serum was then aliquoted into cryovials in 100 μL aliquots and stored at −80 °C until ready for use. Prior to each assay, frozen serum was thawed at room temperature. Serum was diluted 1:5 in assay wash buffer and allowed to equilibrate to room temperature for at least 10 min prior to beginning an assay.

### 2.3. Assay Workflow

Functionalized chips were stored in a desiccator under vacuum until ready for use. First, polystyrene micropillar fluidic cards, which consisted of a sample zone, an initial wicking zone, and a paper strip (Whatman, Little Chalfont, UK) for continuous wicking of the full sample volume, were made hydrophilic by treatment with oxygen plasma (Plasmod Plasma System, Nordson Plasma Systems, Concord, CA) for 1 min. The sensor chip was then attached to the card with 58 μm adhesive tape (3M, St. Paul, MN, USA), with the functionalized sensor surface facing down into the micropillar channel (Figure 1d). Holes were designed in the fluidic cards to allow for light to access the input/output gratings of the chips.

The sensor chip-integrated microfluidic card was then placed on a stage above a custom optical hub (Syntec Optics, Rochester, NY, USA) to allow for vertical alignment of light from a tunable laser source and to a detector (Figure 1e). The grating couplers of the sensor chips were manually aligned to the input/output fibers using an infrared camera and micrometers on the alignment stage, optimizing the power output by adjusting the x/y coordinates of the stage until the power listed on a power meter connected to the output fiber was at a maximum. This could be achieved in 10–30 s. The waveguides were designed for TE polarization, but since the input fiber used was not polarization-maintaining, power through the chip could be further optimized by adjusting polarization paddles attached to the input fiber. The laser was set to scan a 6 nm range, usually around 1552 nm. Once a spectrum was acquired, the laser was set to perform continuous scans, with an approximately 6-second repeat rate.

To start the assay, 20 μL of 20% fetal bovine serum (FBS) was pipetted onto the sample zone of the microfluidic card. As StabilCoat was removed from the surface of the rings, the peaks blue-shifted until the stabilizer was completely removed. Once it was gone, the resonance peaks stabilized and began slowly redshifting as material bound non-specifically to the rings. Once the FBS solution was nearly gone from the sample zone, 30 μL of 20% serum was added. Usually within 1 or 2 measurements, rapid shifting of the antibody (or antigen, depending on the assay) functionalized ring was observed. The sensor was scanned continuously until the sample fluid stopped flowing and dried out, usually after about 5 min.

**Figure 2:**
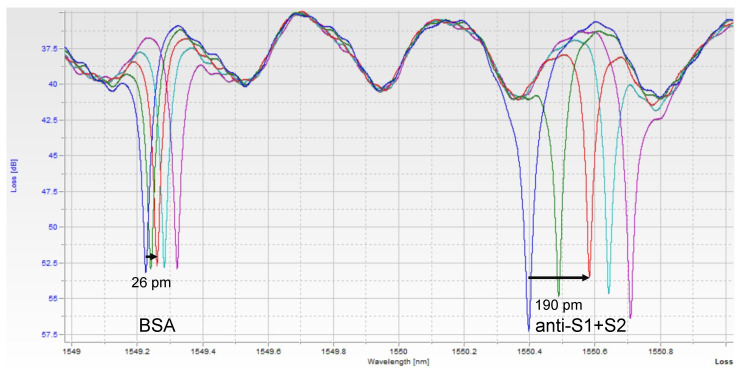
Single Assay Raw Photonic Spectra. Each peak corresponds to one of the ring resonators. As the sample binds to each ring, the resonant wavelength redshifts. The BSA-functionalized (control) ring shifts a small amount due to bulk refractive index changes and non-specific binding of serum components, while the anti-S1+S2-functionalized ring shifts more as spike protein binds to it specifically. The different color spectra here represent increasing timepoints, t = 0, 1, 3, 5, and 7 min. At t = 3 min, the shift of each peak is measured, and the control ring is subtracted from the experimental ring to obtain a relative shift indicative of analyte binding.

Images of the raw data can be seen in Figure 2. Each peak corresponds to one of the 2 rings, with both peaks repeating about every 2 nm, corresponding to the designed free spectral range (FSR) of the resonators. Thus, in a 6 nm scan, usually, 6 peaks were seen, with resonance peaks from the 2 rings repeating 3 times. A custom python script was used to extract peak location over time. Since one ring was functionalized with only BSA, it serves as a control for bulk refractive index changes and non-specific binding. The shift from this control ring was then subtracted from the shift seen in the experimental ring, yielding the relative shift due only to the binding of the target of interest.

## 3. Result

**Figure 3:**
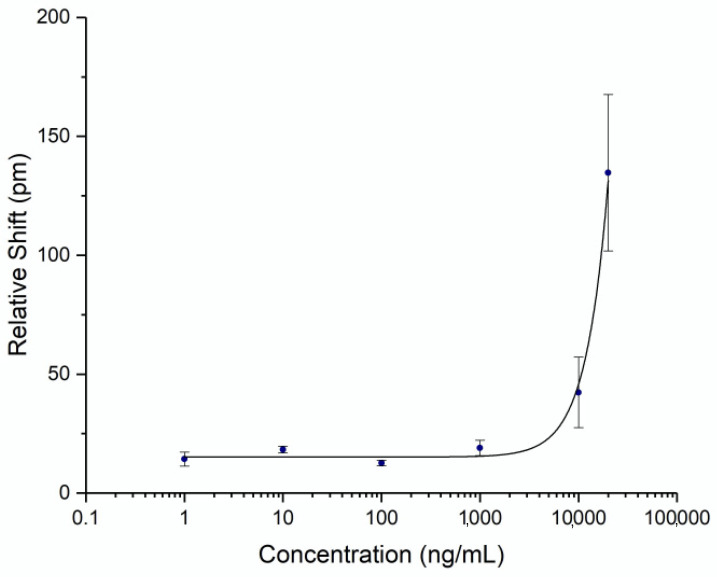
Spike Protein Calibration Curve. Recombinant spike protein S1+S2 ECD was diluted in AWB with 20% FBS to a known concentration and flowed over a chip functionalized with anti-spike antibody. Three assays were run at each concentration. Error bars represent the standard error for each concentration.

To be able to quantify the concentration of spike protein in human subject samples, a titration curve was generated by flowing known concentrations of commercial recombinant whole spike protein S1+S2 ECD (Sino Biological, Wayne, PA, USA) at varying concentrations. Triplicates were performed on separate chips for each concentration to establish error statistics. The calibration curve was fit with a 4-parameter logistic fit and is shown in Figure 3. The unoptimized limit of detection for this assay was determined to be 4.2 μg/mL (2 standard deviations above the noise floor of the assay). While the detection limit was relatively high for a point-of-care assay, it was sufficient for demonstrating an accurate time course of serum spike levels, as discussed below.

**Figure 4:**
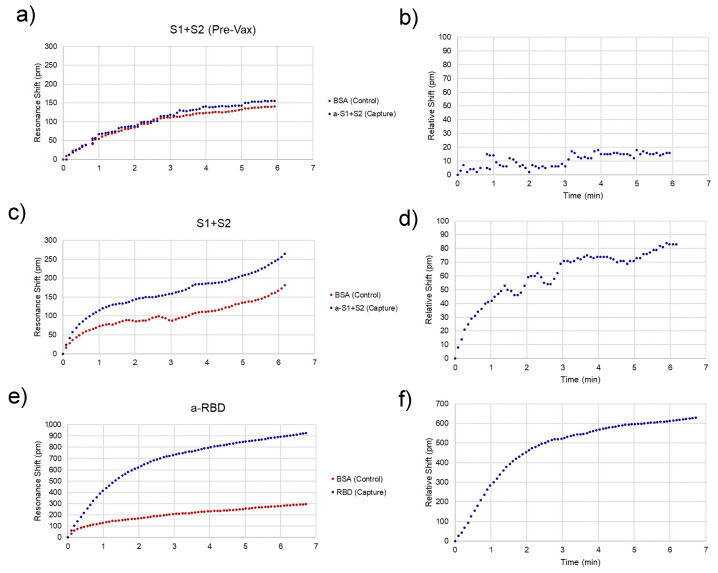
Assay Resonance Traces. Representative peak traces for chips sensing the presence of spike protein in unvaccinated (**a**,**b**) and recently vaccinated (**c**,**d**) subjects and anti-spike antibodies after the second dose. (**e**,**f**). (**a**,**c**,**e**) Each chip has a control (red) and capture (i.e., analyte-specific, blue) ring, whose resonance peaks are tracked over time. (**b**,**d**,**f**) The control ring is subtracted from the capture ring to give the relative shift, which is indicative of specific analyte binding. The relative shift at 3 min is what was recorded to compare between assays.

Representative traces for spike-positive, as well as a pre-vaccination negative control and anti-RBD-positive serum samples are shown in Figure 4. Raw spectra (Figure 2) were analyzed with a Python script described previously [11] and then converted to shifts relative to the position of resonances at time = 0 for both control and experimental rings (Figure 4a,c,e) and relative (BSA ring-subtracted) shift (Figure 4b,d,f). The control ring will shift only as a result of bulk refractive index changes in the sample, temperature fluctuations, and non-specific binding. Thus, the subtracted curve yields an accurate representation of specific antibody/antigen binding over the course of the assay.

While subtracted traces generally provided reliable binding curves, chip-to-chip variability and variations in sample flow across the micropillar substrate result in differences in the time that each assay lasts and possibly the binding kinetics of the assay. The fraction bound at a particular concentration can be extracted from this curve using the equation:

$$fb\left(t,\;c\right)\;=\;fb_{eq}\left(c\right)\times \left[1\;-\;exp\left\{-k_{on}^{obs}\cdot \;t\right\}\right]$$

where *fb* is fraction bound, *t* and *c* are time and concentration, respectively, *fb_eq_* is the fraction bound at equilibrium, *k_on_* is the on-rate, and *k_on_^obs^* is the observable on rate [13]. While this could be used to extract exact concentrations from the raw data, the simplest way to compare concentrations between assays is to simply choose the shift at a particular time point near the end of the sample fluid. In this case, 3 min was chosen as the representative shift for each individual assay for comparison.

**Figure 5:**
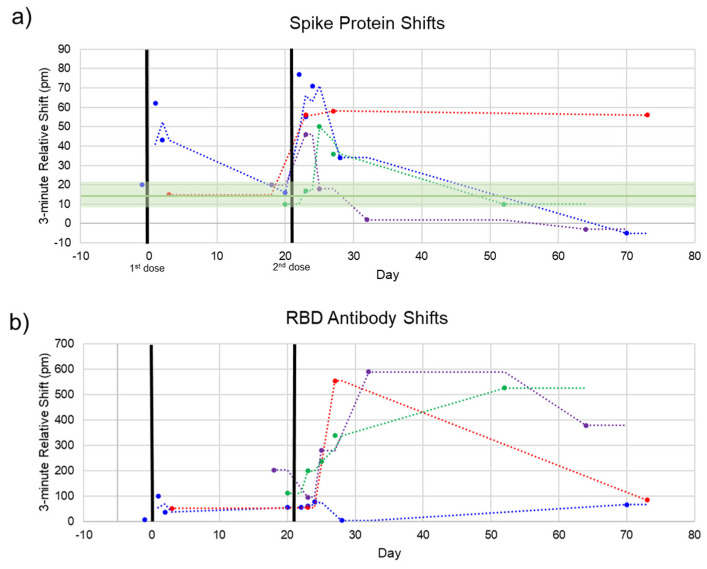
Spike and Anti-Spike Antibody Time Course. Four subjects were repeatedly sampled for serum. Most serum was obtained after the second dose. (**a**) Spike protein rapidly increases after each dose and decreases back to baseline within 1–2 weeks. Pre-vaccination negative control shifts (green line, shaded green = 95% confidence interval) are shown to indicate assay noise. (**b**) Anti-SARS-CoV-2 RBD antibodies over the same time course. In good agreement with clinical trials, our photonic sensors show a modest increase in antibodies after the first dose, reaching high titers after the second dose.

The photonic response data for spike protein is plotted against time in Figure 5, with the two doses marked at Days 0 and 21. The relative shift at 3 min ranged from –5 to 78 pm. Pre-vaccination samples (n = 4) were run to determine the noise of the assay, yielding an average relative shift of 15 pm (95% confidence interval 8.8–21.2 pm), shown as the green line and shaded region in Figure 5a. According to the calibration curve in Figure 3, this corresponds to a maximum concentration of 14.6 μg/mL. Recently published work by Ogata et al. used a Quanterix assay measured spike protein in the blood at concentrations less than 50 pg/mL on a similar timescale [10]. The large discrepancy between our photonic assay and the Ogata work was intriguing and may reflect the early stage of knowledge in this area. While our calibration data were derived from commercial S1 + S2 ECD protein, the protein produced in vivo following BNT162b2 vaccination was prefusion stabilized, membrane-anchored SARS-CoV-2 full-length spike protein [1]. As such, the capture of this protein could bring with it membrane components that would produce a signal in a label-free assay such as ours, but not in a labeled assay. Further differences in structure between commercial and host-generated spikes could result in the quantitative differences observed in this study. Across the four subjects, we observed that there were differences in terms of maximum concentration, time at which the protein concentration peaked, and the time for spike protein to be completely cleared from the blood. Still, the clear trend was a rapid increase in spike protein immediately (1–3 days) after each injection, returning to a baseline level in less than a month. This was in good agreement with the time course reported by Ogata et al. 

Additionally, each sample was assayed for the presence of anti-SARS-CoV-2 antibodies to track the time course of antibody production following vaccination. This response has been well-documented throughout the clinical trials of both mRNA vaccines [1,14] and in our previous work [11]. The pattern we see here is similar, with a modest increase in the first three weeks and a significant boost after the second dose for most subjects. The 3 min shift seen for anti-RBD antibodies ranged from about 75 to 600 pm. A response curve was not generated for antibodies since antibody concentration after vaccination has been well documented. However, due to the similar sizes of antibodies and whole spike protein (~150 kDa for antibodies and 134.6 kDa for S1 + S2), the higher photonic response for antibodies can be attributed to a much higher concentration of circulating antibodies. This is in agreement with our previous study documenting anti-RBD antibodies in convalescent COVID-19 patients, which had antibody concentrations of about 1–150 μg/mL as measured by ELISA, corresponding with photonic resonance shifts of about 50–600 pm [11].

For one subject (Figure 5, red dots), the final timepoint at 73 days was an outlier for both the spike protein and anti-RBD antibody measurements. This subject communicated that they had been sick with a cold concurrent with this final sampling. Thus, these aberrant measurements could be due to increased immune activity in the subject’s bloodstream (for the antibody measurement) and cross-reactivity with the spike protein of a common cold coronavirus such as OC43 [15,16].

## 4. Discussion

Little data exists on the presence of spike protein in circulation following Covid-19 vaccination. Moreover, the degradation of spike proteins in human blood is also poorly studied. Currently, an informational website sponsored by the CDC says that the lifetime of the spike in the bloodstream is “unknown and may be a few weeks,” highlighting the need for studies such as this one [17]. As mentioned earlier, the vaccine contains a sequence for the full-length spike protein, including the transmembrane domain. This poses many questions about the exact mechanism of the immune response, including whether the spike protein is initially only adhered to host cells’ membranes, how it is then cleaved from host cells, and whether/how it enters the bloodstream. This process is poorly studied at this point, but the data shown here demonstrates an important first step in understanding the entire response by confirming that the spike protein generated in response to vaccination does, in fact, enter the bloodstream, persists for over a week, and is completely cleared within one month. Additionally, this suggests that circulating immune cells are involved in the generation of antibodies as well as long-term immunity, though further studies are needed to confirm this. Further, there is almost no data regarding antigen concentrations in the blood following all other types of vaccines, again highlighting the need for a platform that can determine population-scale discernment of vaccine mechanisms, particularly as novel vaccines are rapidly developed in future pandemic situations.

Additionally, this work further highlights the rapid photonic hub and micropillar card combination as constituting a promising platform for diagnostics. While the current sensitivity of the assays described here requires improvement to be broadly useful clinically, we anticipate that much of this can be accomplished given improvements in flow characteristics and reproducibility of the plastic micropillar microfluidics card, given that we have demonstrated highly sensitive assays using identical ring resonator designs and pressure-driven, continuous-flow microfluidics. The small size (<200 μm) of the sensors allows for a small chip footprint and the potential for multiplexing. A multiplex chip that could provide data about viral protein concentrations, antibody concentrations, and even cytokine concentrations in a matter of minutes while maintaining a low cost through wafer-scale production would be a powerful tool for infectious disease diagnostics. The development of this technology will lead to the quicker acquisition of data about disease progression or vaccine response early on in ways that will hopefully help curb future pandemics.

## Data Availability

All original data is available from the corresponding author on request.
